# Quantitative Prediction of Surface Hardness in Cr12MoV Steel and S136 Steel with Two Magnetic Barkhausen Noise Feature Extraction Methods

**DOI:** 10.3390/s24072051

**Published:** 2024-03-23

**Authors:** Xianxian Wang, Yanchao Cai, Xiucheng Liu, Cunfu He

**Affiliations:** Department of information, Beijing University of Technology, Beijing 100124, China; wangxx0902@163.com (X.W.); 13001102233@163.com (Y.C.); hecunfu@bjut.edu.cn (C.H.)

**Keywords:** magnetic Barkhausen noise, surface hardness, MBNHL, BP-NN

## Abstract

The correlation between magnetic Barkhausen noise (MBN) features and the surface hardness of two types of die steels (Cr12MoV steel and S136 steel in Chinese standards) was investigated in this study. Back-propagation neural network (BP-NN) models were established with MBN magnetic features extracted by different methods as the input nodes to realize the quantitative prediction of surface hardness. The accuracy of the BP-NN model largely depended on the quality of the input features. In the extraction process of magnetic features, simplifying parameter settings and reducing manual intervention could significantly improve the stability of magnetic features. In this study, we proposed a method similar to the magnetic Barkhausen noise hysteresis loop (MBNHL) and extracted features. Compared with traditional MBN feature extraction methods, this method simplifies the steps of parameter setting in the feature extraction process and improves the stability of the features. Finally, a BP-NN model of surface hardness was established and compared with the traditional MBN feature extraction methods. The proposed MBNHL method achieved the advantages of simple parameter setting, less manual intervention, and stability of the extracted parameters at the cost of small accuracy reduction.

## 1. Introduction

A mold is a key processing tool for manufacturing parts in machinery manufacturing, electrical machinery, electrical appliances, and other industrial fields. The quality of its raw material, die steel, determines the service life of the mold. Surface hardness is an important indicator of the resistance to external forces and can be used to evaluate the quality of die steel. With the common indentation surface hardness test method, it is difficult to measure the surface hardness of complex parts due to sampling problems and destructive measurements. Fraunhofer IZFP in Germany [1,2,3] developed a 3MA-II versatile micromagnetic testing instrument as an alternative tool to the indentation tester to perform nondestructive evaluation of the surface hardness of ferromagnetic materials.

The microstructures (dislocations, grain boundaries, etc.) of ferromagnetic materials have a pinning effect on the magnetization behavior of magnetic domains (walls) [4,5,6]. In addition, the macroscopic mechanical properties (surface hardness) of grains under externally applied loads are controlled by microstructures and residual stresses [7,8]. In the micromagnetic detection methods of surface hardness, based on the intrinsic correlation between surface hardness, microstructure, and magnetic properties of a material [9,10], a correlation model between magnetic parameters and surface hardness is established by means of the calibration experimental method [11,12,13]. Before determining this correlation, it is necessary to explore the extraction method of micromagnetic parameters and correlation modeling method [14,15].

The performance of the micromagnetic detection method in the nondestructive evaluation of surface hardness has been verified. Nahak et al. [16] used MBN for nondestructive characterization of the surface hardness of electro-discharged machined specimens and found that the peak value and root-mean-square (RMS) value extracted from MBN had a good inverse proportion with surface hardness. Franco et al. [17] reported the approximately linear dependence of magnetic parameters (peak position and derivative of peak height) on the surface hardness of SAE 4140. Liu et al. [18] extracted a total of 11 magnetic features from magnetic Barkhausen noise and tangential magnetic field signals, established a multiple linear regression model and a BP neural network model, which realized the average prediction errors of surface hardness for 12CrMoV (13.7% and 3.7%).

The measurement accuracy of surface hardness with micromagnetic methods depends largely on the stability of magnetic feature signals and the correlation between magnetic features and surface hardness [12,19]. Abundant magnetic features have been extracted from MBN signals using various methods for the characterization of mechanical properties such as surface hardness. In the commonly used MBN processing method, the MBN butterfly curve [20,21] is first plotted, and the peak value, peak position, and other characteristic values are then extracted from the plotted curve to establish the correlation model for the quantitative evaluation of surface hardness. VASHISTA et al. [22] introduced two eigenvalues of counts and events to describe MBN signals, but the features were greatly affected by the artificial selection of the threshold and signal interception time. In addition, many scholars also used the features of MBN signals, including root-mean-square, average amplitude, Barkhausen noise energy, number of counts of the Barkhausen effect impulses [23], and peak-to-width ratio [24] to evaluate the mechanical properties of materials. MBN signals are random noise signals, so the signals collected at different times showed the differences in the peak value and width. Conventional MBN feature extraction methods have some problems, such as complex parameter settings and many manual interventions. Therefore, it is urgent to develop a new feature parameter extraction method to improve the stability of features.

In this study, based on the consideration of the complex parameter setting and frequent manual intervention in the plotting process of MBN butterfly curves and the feature extraction process, a hysteresis loop-shaped MBNHL method was introduced to extract magnetic features. Then, the quantitative prediction of surface hardness of Cr12MoV steel and S136 steel was carried out with self-developed dual-function micro magnetic detection sensors and devices. The MBN butterfly curve and MBNHL curve were plotted with the same set of collected data, and the repeatability of the two methods in extracting magnetic features was evaluated with the coefficient of variation. The magnetic features extracted by the MBNHL method had higher repeatability. Based on the comprehensive consideration of the repeatability of two methods for extracting magnetic features and the performance of magnetic features in surface hardness evaluation, a BP-NN characterization model for surface hardness was established. Compared with the model established by the MBN method, the proposed MBNH method realized the advantages of simple parameter setting, less manual intervention, and stable parameter extraction at the small cost of a slight reduction in prediction accuracy.

## 2. Experimental Device and Preparation of Samples

### 2.1. Experimental Device

**Figure 1:**
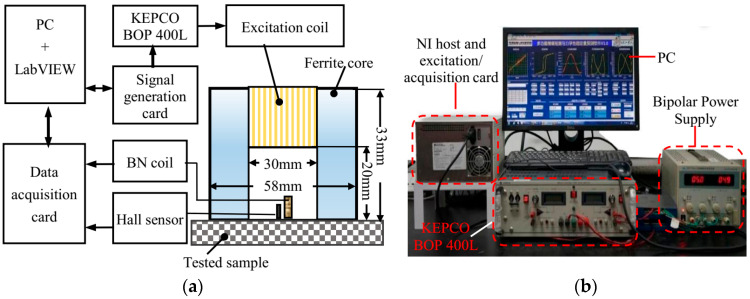
Schematic diagram of MBN detection device. (**a**) configuration of the sensor. (**b**) Picture of the micro-magnetic testing system.

In micromagnetic detection methods, multiple magnetic signals (magnetic Barkhausen noise, tangential magnetic field (TMF), and magnetic incremental permeability (EIP)) are combined together to characterize the mechanical properties of materials [20,25]. In this paper, a sensor (Figure 1a) was developed to simultaneously detect two types of magnetic signals (TMF and MBN). The sensor consisted of a yoke of Fe–Si, an excitation coil, and a detection element (induction coil and Hall element). The low-frequency excitation field was provided by a U-shaped yoke with an excitation coil (0.35 mm in diameter and 300 coil turns) wound at the top, which was made of ferrite with a cross-sectional area of 10 mm × 10 mm. The Hall element (Honeywell SS39E, with a dynamic operating range of ±1000 Gs and a sensitivity of 1.4 mV/Gs) and the induction coil (800 turns in total) in the sensor were used to detect the TMF signal and the MBN signal at the specimen surface, respectively. A Zn–Mn ferrite core (height = 9 mm; diameter = 2 mm) was embedded inside the induction coil to enhance the strength of detected signals.

The experimental device used in this study consisted of a PXI chassis (including a signal generation card and data acquisition card), a power amplifier BOP100-400L, a bipolar power supply, a micromagnetic sensor, and a LABVIEW-based control and analysis software (Figure 1b). The sinusoidal signal (frequency = 12 Hz, amplitude = 8 V) generated by the signal excitation card was first amplified by the BOP power supply and then fed into the excitation coil of the transducer to generate an alternating magnetic field for cyclic magnetization of the specimens. During the cyclic magnetization of the material, the induction coil received the MBN signal, and the Hall element was used to measure the TMF strength change on the material surface. The sampling frequency and the number of sampling points were kept as 1 MS/s and 1 M, respectively. MBN and TMF signals were acquired by the NI PXIe multichannel acquisition card and processed with the LabVIEW 2015 software in the host computer.

### 2.2. Sample Preparation

**Figure 2:**
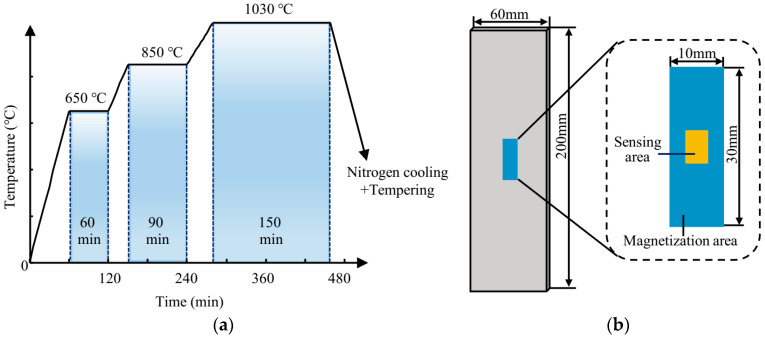
(**a**) Thermal treatment process and (**b**) dimensions of hardness specimens.

In this study, two die steels (Cr12MoV steel and S136 steel in Chinese) were selected to prepare the specimens to be tested. For each material, 60 specimens with the same size (length × width × height = 200 mm × 60 mm × 3 mm) were cut from the steel plate in the rolling direction. Both materials were subjected to the same quenching and tempering processes, and the surface hardness of specimens was adjusted by changing the tempering temperature. The main processes are introduced as follows: Firstly, in the quenching process, all specimens were gradually heated to 1030 °C. Temperature and holding time are shown in Figure 2a. Secondly, in the tempering process, the specimens of each material were divided into 12 batches (5 specimens per batch), and the specimens of Cr12MoV steel and S136 steel were tempered in batches for 210 min in the range of 180 to 720 °C to obtain steel plate specimens with different hardness. The thermally treated specimens were polished at the center (Figure 2b). The MBN and TMF signals of the two materials (a total of 120 specimens) were first tested with a micromagnetic testing system, and then the surface hardness of specimens was tested with a Vickers hardness tester (30 HV with a 30-kg load). The micromagnetic testing and Vickers hardness measurements were carried out in the same magnetized area (Figure 2b).

**Figure 3:**
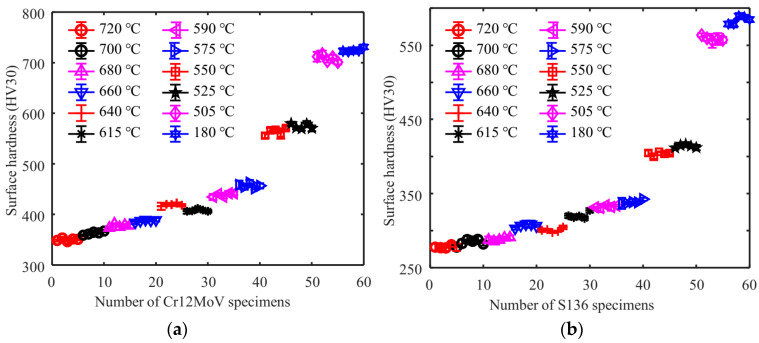
Test results of tempering temperature and surface hardness of (**a**) Cr12MoV and (**b**) S136.

The entire specimen was placed in the furnace for heat treatment, so each specimen was assumed to have a uniform hardness. After testing the magnetic signals of all samples, a microhardness tester (HV30 with a load of 30 kg) is used to test the Vickers hardness value of the specimen surface. It is worth noting that the microhardness test position should be consistent with the magnetic signal test position. To ensure the accuracy and reliability of the test results, three tests were conducted at close positions on the specimen, and the average value was calculated. The average values of the three tests are shown in Figure 3. The surface hardness of Cr12MoV and S136 steels fluctuated within the ranges of 350 HV to 750 HV and 250 HV to 600 HV. The five specimens in the same batch had similar surface hardness with slight differences.

### 2.3. MBN Detection Experiments

To evaluate the repeatability of the MBN and TMF signals, 60 specimens of each material were tested multiple times. For a single specimen, 15 sets of repeated tests with MBN and TMF signals were performed at the same location. A demagnetization device was used to demagnetize the specimens before each test, and a Gauss meter was used to measure the residual magnetic strength to ensure that the initial magnetic state of the samples remained unchanged. Upon the completion of the tests, 15 × 60 = 900 sets of magnetic signals were obtained for each material.

## 3. Processing Methods of MBN Signals

The stability of the magnetic features used as model inputs is significant in the construction of an accurate surface hardness model. MBN signals are random, so stable MBN features are conducive to constructing a high-precision model.

### 3.1. MBN Butterfly Curve

**Figure 4:**
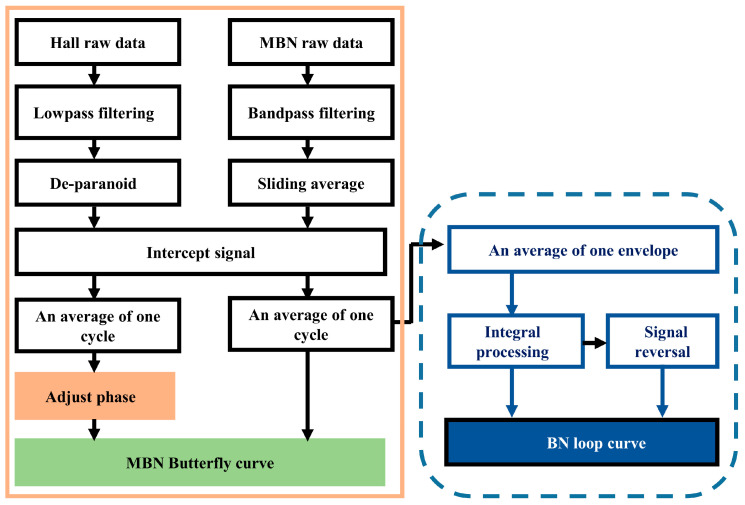
Flow chart of plotting the BN butterfly curve and BN loop curve.

The MBN butterfly curve describes the variation of the magnetic Barkhausen noise envelope with the tangential magnetic field strength waveform within one cycle. Plotting the MBN butterfly curve is a common way to extract MBN features. The plotting process of the MBN butterfly curve is shown in Figure 4 and includes signal filtering (filter type, high/low cutoff frequency, order, etc.), signal interception, and curve plotting.

**Figure 5:**
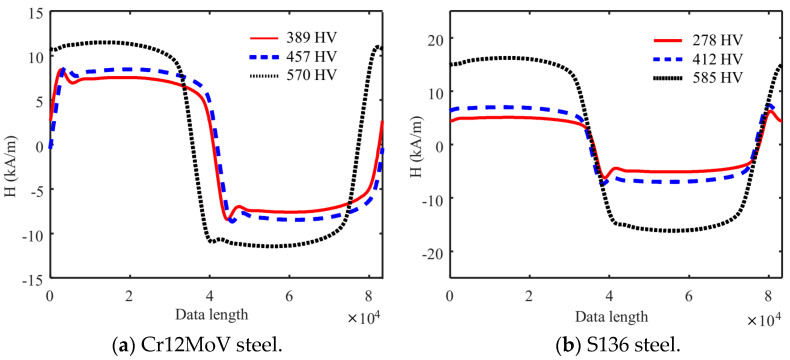
TMF signals for different surface hardness specimens.

In the first step, TMF signals were filtered. The raw signals measured by the Hall sensor were superimposed with high-frequency interference noise, which should be removed with a low-pass filter. A Butterworth low-pass filter (cutoff frequency of 200 Hz) was selected to filter the signals. After the DC bias was removed, the TMF signal was obtained through the calculation with Equation (1). Figure 5a and Figure 5b, respectively, show the TMF signals measured in different hardness specimens of Cr12MoV and S136 steel. The TMF signals are affected by the hysteresis characteristics in the tested specimens, and the TMF waveforms of different specimens are different.

(1) $$H=\frac{V}{{k\times \mu }_{0}},$$

where *V* is the Hall voltage after subtracting the bias; *k* is the conversion coefficient of the Hall sensor and equals 1.5 mV/G; and *µ*_0_ is the permeability of vacuum and equals 4 × π × 10^−7^.

**Figure 6:**
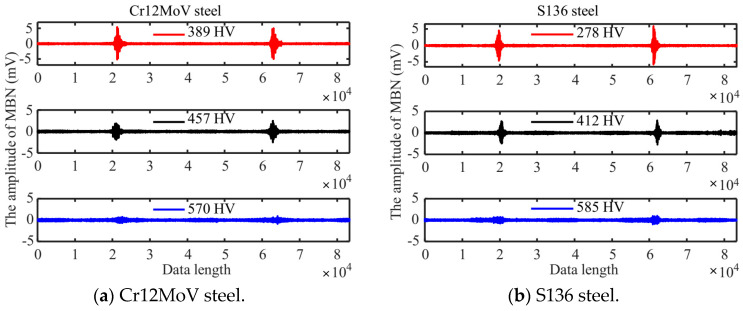
MBN signals of different surface hardness specimens.

In the second step, MBN signals were filtered. The MBN signal is a random electromagnetic noise signal. During signal reception, the detection coil outputs a wide-band signal, so the filtering process of detection signals is required. The correct choice of filtering frequency is crucial for post-processing the noise signal. In this study, a 4-th order Butterworth band-pass filter (filter frequency band 30 kHz to 90 kHz) was used to filter the signals detected with the receiver coil. Typical MBN signals measured in the different surface hardness specimens of Cr12MoV steel and S136 steels are shown in Figure 6a,b. Both the waveforms and peaks of MBN signals are affected by surface hardness, which essentially suggests microstructural change.

In order to extract the features of MBN, the filtered MBN signals were processed by sliding average. The window size *n* was selected for the sliding average and the root-mean-square (RMS) operation was performed with the data in the window. Starting from the first data point, the operation then slid to the next data point in sequence. The smoothness of the MBN envelope is determined by the value of *n* and should be determined according to the actual situation. Based on prior experience, in this paper, an *n* = 400 was selected to perform four repetitions of sliding RMS operation in order to obtain a smooth MBN signal envelope.

In the third step, signals were intercepted. In the process of magnetic signal testing, the initial magnetization and remnant magnetization state of the material interfered with the obtained signals, and the whole cycle interception of the filtered TMF and MBN signals could eliminate the interference. In this paper, the 4th maximum point of the TMF signal was selected as the starting point, and eight cycles of the TMF signal and MBN signal were intercepted and retained synchronously.

**Figure 7:**
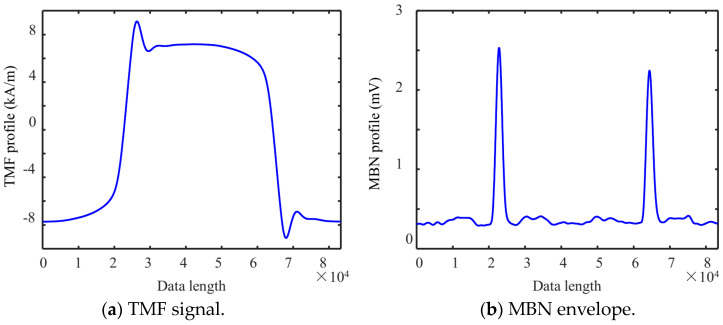
One cycle magnetic signal.

In the fourth step, the MBN butterfly curve was plotted. The micromagnetic signals are related to the magnetization process of magnetic domains, which is a random process. The magnetic features measured in multiple magnetization cycles had random fluctuations. The intercepted eight-cycle TMF and MBN signals are simultaneously processed with an average envelope to obtain one-cycle TMF and MBN signals (Figure 7). The magnetic Barkhausen noise butterfly curves were plotted with the signal envelopes (Figure 7a,b) as horizontal and vertical coordinates, respectively.

In the process of filtering and sliding the average of TMF and MBN signals, a phase shift of the signals was inevitable. The magnitude of the phase shift directly affected the shape of the plotted butterfly curve and magnetic features. Generally, MBN appeared at the moment of TMF reversal. During signal processing, time-shift adjustment of TMF signals was performed to improve the quality of the MBN butterfly curve. At present, phase adjustment between TMF signals and MBN envelope is mainly performed based on personal experiences and parameter adjustment is not introduced in related theories or standards.

**Figure 8:**
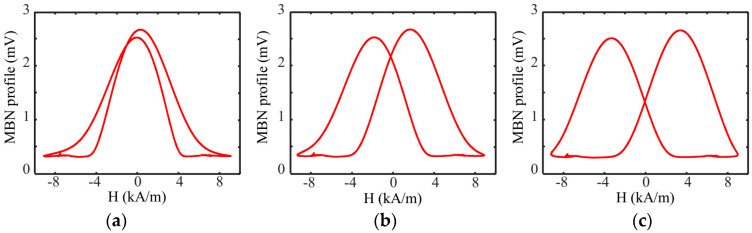
Butterfly-like MBN curves of different time-shift adjustments: (**a**) 0 cycle; (**b**) 1/160 cycle; and (**c**) 1/80 cycle.

Figure 8 illustrates the butterfly curve obtained after different time-shift adjustments from the same data set. In this paper, the time-shift adjustment was performed in such a way that the peak of the MBN envelope was close to the over-zero position of TMF. Table 1 lists the magnetic features and coefficient of variation σ (σ = standard deviation/mean value) extracted from the butterfly curves through different time-shift adjustments. A small coefficient of variation indicated good repeatability and stability of the signals in repeated experiments. A large coefficient of variation indicated that the system had poor repeatability detection performance for magnetic covariates, which was not conducive to the high-precision quantitative characterization of surface hardness. The coefficients of variation of the magnetic parameters (*Hcm*, *DH75M*, and *DH50M*) extracted from the butterfly curves respectively reached 38.8%, 23.1%, and 14.6% when the phase was not adjusted (Table 1). When the time-shift adjustment was performed within about 1/80 cycle, the coefficients of variation of the repeated detection data of the above magnetic parameters reduced to 5%. For the actual detection signals in this paper, a time-shift adjustment of the 1/80 cycle was chosen.

**Figure 9:**
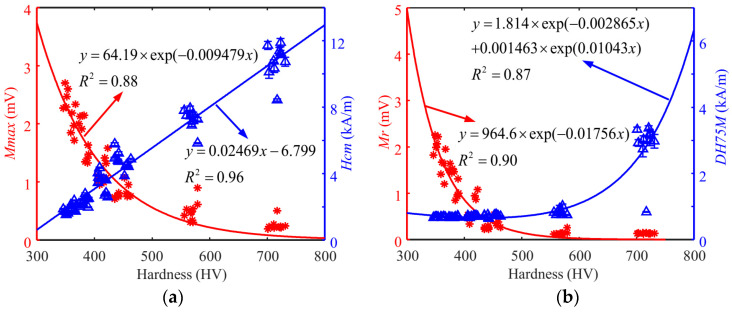
Dependency of the features on the surface hardness of Cr12MoV steel. (**a**) The relationship between *Mmax* (*Hcm*) and hardness. (**b**) The relationship between *Mr* (*Hcm*) and hardness.

**Figure 10:**
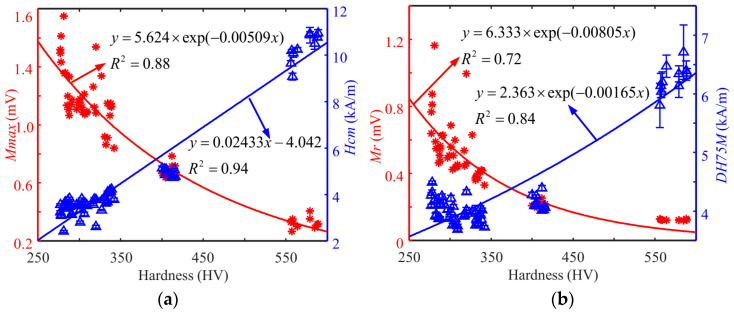
Dependency of the features on the surface hardness of S136 steel. (**a**) The relationship between *Mmax* (*Hcm*) and hardness. (**b**) The relationship between *Mr* (*DH75M*) and hardness.

After the parameter setting was determined during signal processing, the magnetic features obtained from the surface hardness tests of Cr12MoV steels and S136 steels (60 specimens for each steel) were analyzed. The average values of the coefficients of variation for the two materials are listed in Table 1. The *DH50M* coefficient of variation was greater than 5%, so it was not used in subsequent surface hardness modeling. Figure 9 and Figure 10 show the dependency of typical magnetic signatures on the surface hardness of the specimens of Cr12MoV steel and S136 steel. The same magnetic features of Cr12MoV and S136 steels showed a similar trend with surface hardness. *Mmax* shows a decreasing trend with the increase in surface hardness, whereas *Hcm* values showed the opposite trend. The difference might be interpreted as follows: The specimens with high surface hardness had a high percentage of martensitic volume content, which had a strong pinning effect on magnetic domains. Therefore, the flip of magnetic domains in the specimen required more energy from the applied magnetic field. 

### 3.2. Calculation of MBN Hysteretic Curve

In the plotting process of MBN butterfly curves, artificial time-shift adjustment is required and characterized by complicated parameter settings and many manual interventions. In this paper, we proposed a new processing method for MBN signals, in which the envelope of MBN signals was integrated to obtain the hysteresis loop-shaped MBNHL curve and then new features were extracted to determine their relationship with surface hardness.

**Figure 11:**
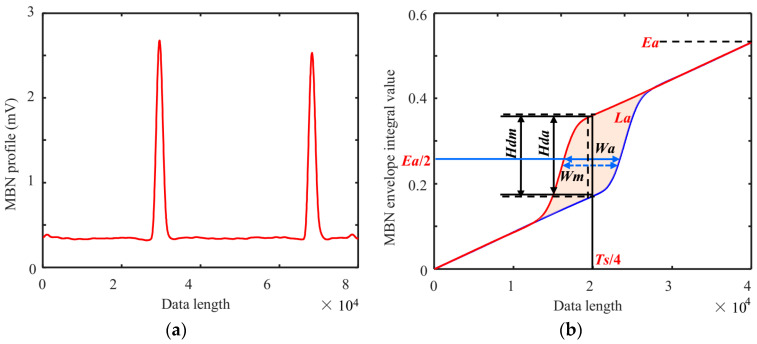
(**a**) MBN envelope obtained after resampling and (**b**) MBNHL curve.

The plotting steps of the MBNHL curve are shown in Figure 4. After the MBN signal processing steps of band-pass filtering, sliding average, and signal interception were completed according to Step 1 to Step 3 in Section 3.1, the MBN envelope was integrated and calculated. Firstly, the MBN envelope was averaged over eight magnetization cycles to obtain the envelope curve for a single magnetization cycle (Figure 11a). Then, with the first point of the single cycle as the starting point, the envelope of the first half cycle was integrated, and the envelope of the second half cycle was integrated and flipped. Finally, a closed curve was obtained through the envelope integration of the first half cycle and the envelope integration and flipping of the second half cycle. The MBNHL curve was obtained by integrating the MBN signal envelope so that the MBNHL feature corresponded to the MBN feature. For example, the maximum slope of MBNHL was the peak value of the MBN signal, *Mmax*, and the location where the maximum slope occurred was the peak location, *Hcm*. In addition to the features mapped by the MBN envelope, new features shown in Table 2 could be extracted based on the MBNHL curve.

**Figure 12:**
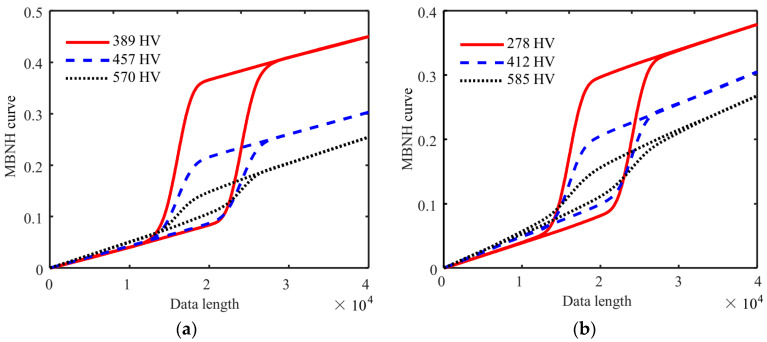
MBNHL curves for different surface hardness specimens of (**a**) Cr12MoV steel and (**b**) S136 steel.

Typical MBNHL curves plotted for different surface hardness specimens of Cr12MoV steel and S136 steel are shown in Figure 12a,b. The MBNHL curve could be used to distinguish the hardness variation and was sensitive to surface hardness. The coefficients of variation of the six features extracted from the MBNHL were less than 2% (Table 2), indicating the highly stable magnetic features extracted by the MBNHL method.

**Figure 13:**
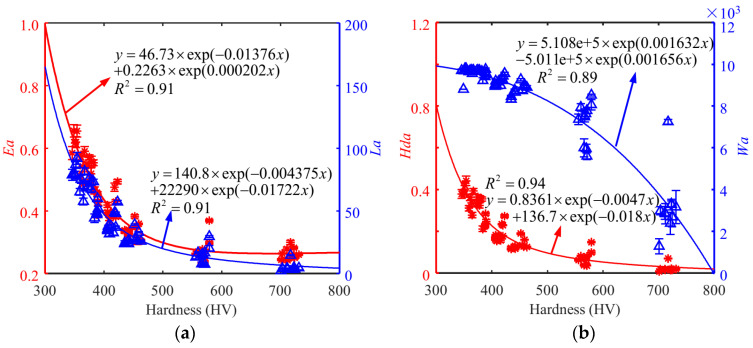

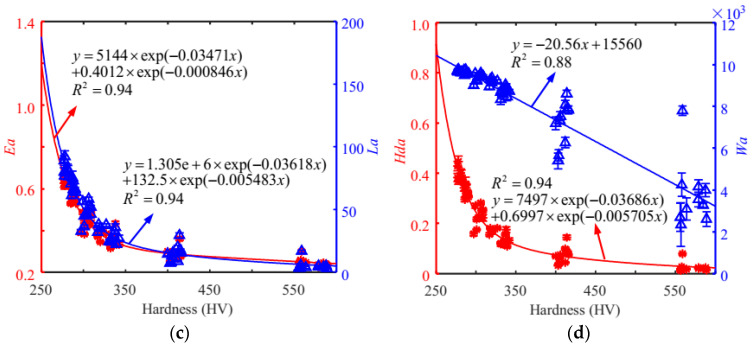
Dependency of the features of *Ea*, *La*, *Hda*, and *Wa* on the surface hardness of (**a**,**b**) Cr12MoV steel and (**c**,**d**) S136 steel.

Figure 13 shows the correlation between MBNHL features and surface hardness. The peak and area of MBNHL curves for both materials showed a decreasing trend with the increase in surface hardness. It is worth noting that the changing trend of one magnetic feature with surface hardness is the same for both materials and shows a nonlinear correlation.

The key to the quantitative prediction of surface hardness with the micromagnetic method is to provide stable and reliable input data. Compared with the traditional MBN butterfly curve method, the waveform processing and features extraction method of the MBNHL curve showed the advantages of simple parameter setting, less manual intervention, and stable data.

## 4. Results and Discussion

The mechanism of microstructure influence on magnetic domain motion has not been well explained [26], so it is not possible to establish an accurate analytical model between the microstructure of the material and the surface hardness [27]. Therefore, a new method of micromagnetic nondestructive measurement of mechanical properties is developed to obtain the macroscopic magnetic feature signals and mechanical properties of materials step by step through calibration experiments and to establish quantitative prediction models of mechanical properties using artificial intelligence algorithms. With the multifunctional micromagnetic inspection instrument, abundant magnetic features that were sensitive to surface hardness were obtained in Section 3. Considering the nonlinear relationship between most of the magnetic features and surface hardness and the excellent performance of neural networks in solving nonlinear problems, a neural network prediction model of surface hardness was developed with several sensitive magnetic parameters as the input to characterize surface hardness in the study.

**Figure 14:**
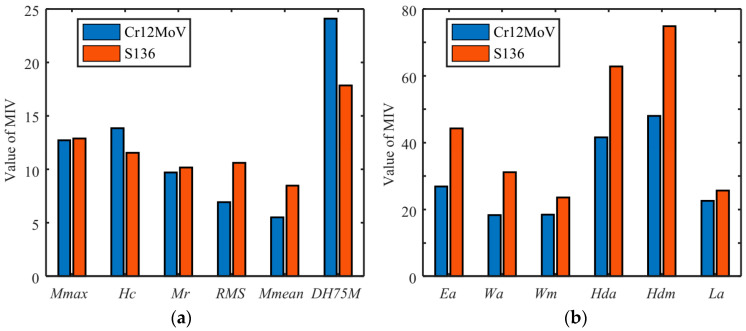
Analysis results of MIV weight coefficients with the data from (**a**) MBN and (**b**) MBNHL.

The selection of input parameters directly affects the performance of a BP neural network model. The mean influence value (MIV) method can be used to evaluate the weight of the influence of each parameter of the input neural network on output variables. A BP neural network with a three-layer structure (input, hidden, and output layers) was chosen to find the MIV value for each magnetic parameter of the input model, where the number of neurons in the middle-hidden layer was fixed at 10. The absolute value of MIV represented the degree of influence of each parameter in the model. Figure 14 shows the MIV analysis results of both materials.

**Figure 15:**
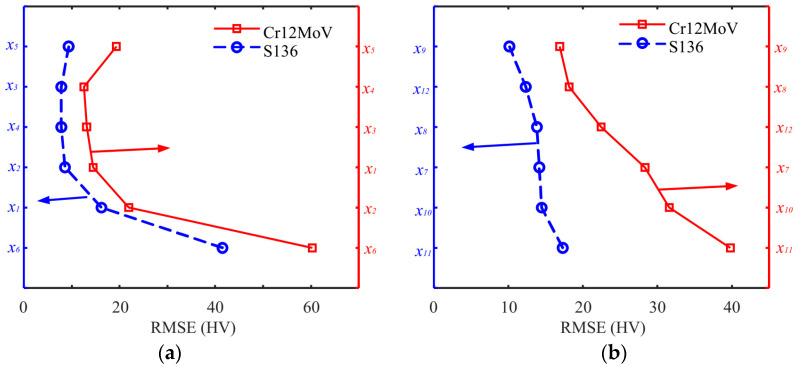
RSME values of the BP model with the input data from (**a**) MBN and (**b**) MBNHL.

The features were input into the model in descending order of MIV weight coefficients, and the number of input nodes for the BP model was determined with the training data for internal validation and RMSE estimation of the BP model. Figure 15 shows the RMSE results of the BP model with different input nodes. The six magnetic features calculated with the MBN method were input into the model in descending order of weighting coefficients. The RMSE decreased significantly when the number of input nodes was increased from one to five in the models of Cr12MoV and S136 steels (Figure 15a). RMSE increased when the number of input nodes increased to six (Figure 15a). Therefore, parameters *x*_1_, *x*_2_, *x*_3_, *x*_4_, and *x*_6_ were selected and used in the final training phase of the BP model. The same analysis was performed for the six magnetic features obtained with the MBNHL method (Figure 15b). The RMSE of the model decreased with the increase in input features, and the input nodes of the final prediction model for Cr12MoV and S136 steels were *x*_7_ to *x*_12_.

A BP neural network was used to construct a quantitative prediction model of surface hardness after the input parameters and nodes were identified. Both the training and validation sets were normalized before they were input into the BP neural network model. The predicted values produced by the model were then subjected to reverse normalization. Based on the neural network toolbox (nntool) in the Matlab 2020b software, the activation functions of the hidden layer and output layer were, respectively, the “tansig” function and the “purelin” function, and the “trainlm” training function. The chosen magnetic parameters were selected as input nodes, and surface hardness was the output node.

**Figure 16:**
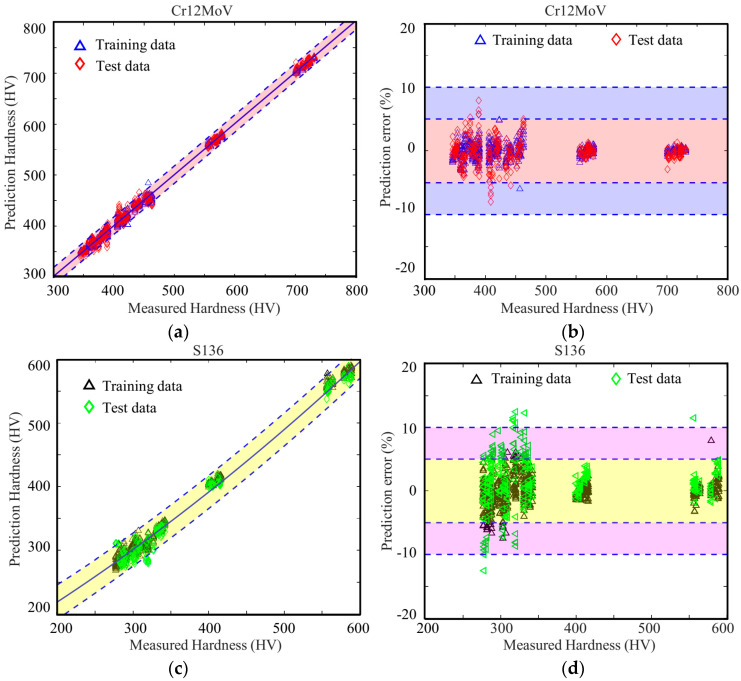
Prediction errors of the models based on the parameters obtained with the MBN method for (**a**,**b**) Cr12MoV steel and (**c**,**d**) 3Cr13 steel.

In order to train the model, two-thirds of the magnetic parameters derived from MBN for Cr12MoV steel and S136 steel were randomly chosen as the training set (600 sets of data), and the remaining one-third of the data (300 sets of data) was utilized as the validation set to assess the prediction accuracy of the model. Figure 16 displays the prediction errors of surface hardness of Cr12MoV steel and S136 steel. For the two materials, the average prediction errors of surface hardness were 1.4% and 3.25%, respectively. The largest errors were, respectively, 8.0% and 12.5%. Among the 300 cases, only nine of the S136 data points had a prediction error of more than 10%.

**Figure 17:**
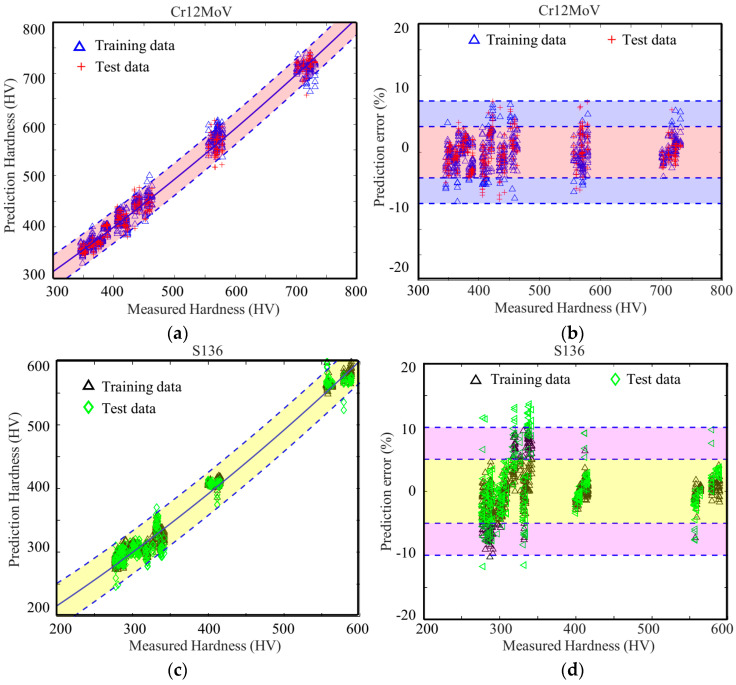
Prediction errors of the models based on parameters obtained with the MBNHL method for (**a**,**b**) Cr12MoV steel and (**c**,**d**) 3Cr13 steel.

When the network structure and model parameters of the BP model were fixed, 2/3 (600 sets of data) of the magnetic parameters generated by MBNHL were randomly chosen as the training set, and the remaining 1/3 data set (300 sets of data) were used to assess the prediction accuracy of the model. The average errors of surface hardness of Cr12MoV steel and S136 steel were, respectively, 2.5% and 3.8% (Figure 17). The maximum errors of Cr12MoV steel and S136 steel were, respectively, 9.9% and 13.7%. In the 300 cases of Cr12MoV steel and S136 steel, the prediction errors of 22 cases of S136 steel were larger than 10%.

## 5. Conclusions

In this study, BP-NN and micro-magnetic testing were combined together to achieve a quantitative prediction of the surface hardness of S136 and Cr12MoV steels. The phase adjustment in the curve plotting process had a significant impact on the shape of the plotted butterfly curve and the stability of feature extraction. The generally used MBN butterfly curve plotting method is easily influenced by personnel experiences. A plotting and parameter extraction of a hysteresis loop-shaped BN circular curve was proposed in order to address the problems of complex parameter setting and excessive user intervention in the plotting process of MBN butterfly curves. Surface hardness and the newly derived features exhibited a strong association. Compared with traditional MBN feature extraction methods, the new features exhibited high stability.

For the purpose of making a quantitative prediction of the surface hardness of Cr12MoV steel and S136 steel, a BP-NN model with the same structural parameters was established. For Cr12MoV steel and S136 steel, the average prediction errors of the features derived by MBN were about 1.4% and 3.25%, respectively. The average prediction errors of the features extracted with the MBNHL method were 2.5% and 3.8%, respectively. Compared to the MBN method, the proposed MBNHL method is characterized by simple parameter configuration, reduced user intervention, simple parameter extraction, and comparable prediction accuracy.

## Figures and Tables

**Table 1:** Coefficients of variation of conventional features extracted from the MBN profile.

Features	MBN Signals	0	1/160	1/80	Cr12MoV	S136
*x*_1_	Peak height of the MBN butterfly curve, *Mmax*	3.9%	3.9%	3.9%	3.1%	3.5%
*x*_2_	Peak position of the MBN butterfly curve, *Hcm*	38.8%	3.7%	1.5%	1.3%	2.1%
*x*_3_	Intercept of MBN envelope at the vertical axis, *Mr*	3.9%	3.9%	4.1%	3.9%	3.4%
*x*_4_	Mean value of MBN envelope, *Mmean*	3.9%	3.9%	3.8%	3.1%	2.9%
*x*_5_	Full width at 75% of maxima of MBN butterfly curve, *DH75M*	23.1%	3.5%	1.9%	2.0%	2.4%
*x*_6_	Full width at 50% of maxima of MBN butterfly curve, *DH50M*	14.6%	3.1%	1.5%	15.4%	9.1%

**Table 2:** Features extracted from the MBNHL and coefficient of the variation.

Features	MBNHL Curve Explanation	Coefficient of Variation (%)	
		Cr12MoV	S136
*x*_7_	Maximum point on the integration curve, *Ea*	1.9	1.5
*x*_8_	Time difference between two points on the integration curve corresponding to Ea/2, *Wa*	1.1	1.1
*x*_9_	Maximum time difference for the same integral value on the integration curve, *Wm*	1.6	1.2
*x*_10_	Height difference between the two integration curve at Ts/4, *Hda*	3.5	3.5
*x*_11_	Maximum difference between two integration curves at the same time, *Hdm*	3.2	3.2
*x*_12_	The area of the closed curve, *La*	3.6	3.9

## Data Availability

Data are contained within the article.
